# Synthesis and Characterization of Ibuprofen–TiO_2_ Functionalized PCL Biomembranes as Candidate Materials for Wound Dressing Applications

**DOI:** 10.3390/bioengineering13010092

**Published:** 2026-01-13

**Authors:** Jael Adrian Vergara-Lope Nuñez, Amaury Pozos-Guillén, Marine Ortiz-Magdaleno, Israel Alfonso Núñez-Tapia, Silvia Maldonado Frias, Marco Antonio Álvarez-Pérez, Febe Carolina Vazquez-Vazquez

**Affiliations:** 1 Laboratorio de Inmunoquímica I, Departamento de Inmunología, Escuela Nacional de Ciencias Biológicas, Instituto Politécnico Nacional, Prolongación de Carpio y Plan de Ayala s/n, Santo Tomás, Ciudad de México 11340, Mexico; adrianvelonu@gmail.com; 2 Tissue Bioengineering Laboratory, DEPeI-FO, National Autonomous University of Mexico, Circuito Exterior s/n, Ciudad Universitaria, Ciudad de México 04510, Mexico; sylvymaf@yahoo.com.mx; 3 Laboratory of Basic Sciences, Faculty of Dentistry, San Luis Potosí University, San Luis Potosí 78210, Mexico; apozos@uaslp.mx (A.P.-G.); marine.ortiz@uaslp.mx (M.O.-M.); 4 Instituto de Investigaciones en Materiales, Universidad Nacional Autónoma de México, Circuito Exterior s/n, Ciudad Universitaria, Ciudad de México 04510, Mexico; isrant86@gmail.com; 5 Laboratorio de Materiales Dentales, DEPeI, School of Dentistry, Universidad Nacional Autónoma de México, Circuito Exterior s/n, Ciudad Universitaria, Ciudad de México 04510, Mexico

**Keywords:** wound dressing coverage, polycaprolactone, titanium dioxide nanoparticles, ibuprofen functionalization, antimicrobial properties, tissue engineering applications

## Abstract

Wound dressing coverages (WDC) play a key role in protecting skin lesions and preventing infection. Polymeric membranes have been widely explored as WDC due to their ability to incorporate bioactive agents, including antimicrobial nanoparticles and non-steroidal anti-inflammatory drugs (NSAIDs). In this study, polycaprolactone (PCL)-based membranes functionalized with titanium dioxide nanoparticles (TiO_2_ NPs) and ibuprofen (IBP) were fabricated using a film manufacturing approach, and their structural and biocompatibility profiles were evaluated. The membranes were characterized by SEM, FTIR and XPS. Bands at 1725 cm^−1^, 2950 cm^−1^, 2955 cm^−1^, 2865 cm^−1^ and 510 cm^−1^ proved molecular stability of reagents during manufacture. In SEM, the control shows the flattest surface, while the PCL-IBP and PCL-IBP-TiO_2_ NPs groups had increased rugosity. In vitro biocompatibility was evaluated using human fetal osteoblasts (hFOB). On day 3, the cell adhesion response of hFOB seeded in PCL-IBP and PCL-IBP-TiO_2_ NPs groups showed the biggest absorbances (*p* = 0.0014 and *p* = 0.0491, respectively). On day 7 PCL-IBP group had lower lectin binding than the control (*p* = 0.007) and the PCL-IBP-TiO_2_ NPs (*p* = 0.015) membranes, but no evidence of cytotoxicity was observed in any group. Furthermore, the Live/Dead test adds more biocompatibility evidence to conveniently discriminate between live and dead cells. The PCL polymeric membrane elaborated in this study may confer antiseptic, analgesic and anti-inflammatory properties, making these membranes ideal for skin lesions.

## 1. Introduction

The skin plays a crucial role in protection against environmental threats. When this barrier is disrupted, a complex biological process is initiated to restore its protective function [1,2]. The development of devices that enhance skin regeneration is therefore of significant interest, particularly for wounds associated with chronic conditions such as diabetes mellitus, vascular diseases, or prolonged mechanical pressure in critically ill patients [1,3,4].

Wound dressing coverages (WDC) are commonly applied to protect damaged skin [5], prevent infection [3], reduce pain, and promote reepithelialization [6]. In addition, WDC should be fabricated from biodegradable and biocompatible materials to ensure safe interaction with biological tissues [7]. A wide range of dressing strategies has been developed, including hydrocolloids, foam dressings, biological wound dressings, hydrogels, and polymeric films [8,9].

Synthetic biomaterials remain the primary resource for WDC fabrication due to their thermal stability, favorable mechanical properties, and ability to be readily loaded with therapeutic agents [10,11]. These properties may be exploited individually or synergistically. Accordingly, WDC have been functionalized with drugs or bioactive molecules to enhance epithelial cell migration, proliferation, and adhesion, as well as to prevent microbial infection during the wound healing process [12,13].

Polymeric membranes offer versatile control over physicochemical properties relevant to wound healing applications [14]. By adjusting parameters such as membrane flexibility, absorption, and desorption, WDCs can modulate drug release kinetics or provide moisturizing effects. Among the polymers commonly used, polycaprolactone (PCL) has attracted considerable attention and has been extensively employed in the fabrication of membranes for skin wound healing due to its biocompatibility and mechanical stability [15,16].

The control of wound-related infections remains a major clinical challenge. To address this issue, metallic nanoparticles (NPs) and antimicrobial macromolecules have been incorporated into WDC [17,18]. In particular, nanoparticles such as silver, zinc oxide, titanium dioxide (TiO_2_), and copper have received significant attention owing to their antimicrobial activity, high stability in biological environments, and generally favorable effects on the wound healing process [19,20].

WDC can also be functionalized with drugs to modulate the inflammatory response. The localized delivery of non-steroidal anti-inflammatory drugs (NSAIDs) provides analgesic and antipyretic effects, thereby reducing pain and inflammation at the wound site. Ibuprofen (IBP), a widely used NSAID, inhibits cyclooxygenase (COX), an enzyme highly expressed in skin tissue [21]. This inhibition prevents the conversion of arachidonic acid into prostaglandins and thromboxanes, resulting in a reduction in inflammatory mediators and subsequent attenuation of pain receptor activation. Localized drug delivery minimizes systemic exposure and associated side effects, making drug-loaded WDCs an attractive strategy for controlling inflammation and improving patient comfort [22,23].

The use of PCL and TiO_2_ has been previously reported. This combination has been employed to fabricate fibrillar membranes for the controlled release of tetracycline [20], as well as to produce films incorporating cerium dioxide and chitosan to enhance the antibacterial performance of TiO_2_ [18]. However, these manufacturing approaches typically require the use of high-voltage electric fields or elevated temperatures to synthesize the wound dressing coverages and may degrade the functionalized molecules used [20,24].

Although PCL-based systems, antimicrobial nanoparticles, and anti-inflammatory drugs have been independently explored for wound healing applications, their integration into a single platform using a rapid and straightforward film manufacturing approach remains limited. For this reason, the objective of this study was to evaluate the structural characteristics and cellular response of a PCL-based WDC functionalized with TiO_2_ NPs and IBP. The proposed device was therefore designed to combine components with well-documented antimicrobial and anti-inflammatory potential, establishing its biocompatibility profile as a foundation for future multifunctional wound healing applications.

## 2. Materials and Methods

### 2.1. Membrane Manufacturing

The polymeric membranes were fabricated following a previously reported method, with modifications in the polymer composition [25]. Briefly, PCL pellets (polycaprolactone, M_W_ = 70,000–90,000 g/mol; Sigma-Aldrich) were dissolved in a solvent mixture of acetone and chloroform (3:1, respectively) to obtain a final polymer concentration of 6% (p/v). The resulting stock solution was divided into three experimental groups. The first group consisted of pure PCL and was used as the control. The second group was supplemented with ibuprofen (PCL-IBP), while the third group contained ibuprofen and titanium dioxide nanoparticles (PCL–IBP–TiO_2_ NPs).

For the PCL-IBP formulation, ibuprofen (M_w_ = 206.28 g/mol, CAS No: 15687-27-1 Merck) powder was added at a concentration of 2%. In the PCL-IBP-TiO_2_ NPs group, 2% of ibuprofen and 0.3% of TiO_2_ NPs (M_w_ = 79.87 g/mol, Sigma-Aldrich) were incorporated in the stock solution. All solutions were magnetically stirred at room temperature for 2 h to ensure complete homogenization. Subsequently, the solutions were cast into Petri dishes and allowed to dry at room temperature for 2 h. The resulting membranes were sterilized under a germicidal UV lamp (ZW30S19W-Z894, diameter 19 mm, 30 W potency, 110 V, 107 μw/cm^2^ irradiance) under the laminar flow cabinet for 15 min before further characterization and biological evaluation.

### 2.2. Membrane Characterization

The membrane samples were dehydrated using a graded ethanol series and subsequently sputter-coated with a 5 nm gold layer (EMS 150R, Quorum, East Sussex, UK). Surface morphology was examined using scanning electron microscopy (SEM, JSM-6700, JEOL, Tokyo, Japan) operated at an accelerating voltage of 10 kV. SEM images were acquired at magnifications of 50×, 100×, and 500×.

Molecular characterization was performed by Fourier transform infrared (FTIR) spectroscopy (Thermo Scientific Nicolet 6700, Thermo Fisher Scientific, Waltham, MA, USA) using the attenuated total reflectance (ATR) mode. Spectra were recorded in the range of 4000 cm^−1^ to 400 cm^−1^ with a spectral resolution of 4 cm^−1^. Additionally, crystalline phase analysis was conducted by X-ray (XRD) using a diffractometer operating in Bragg–Brentano geometry with Cu-Kα lamp (λ = 1.5417 Å) over a range of 2θ = 5° to 2θ = 40°.

### 2.3. In Vitro Studies

The human fetal osteoblast cell line (hFOB, ATCC CRL-11372) was used for biological evaluations. hFOB cells were cultured in 75 cm^2^ culture flasks with Dulbecco’s Modified Eagle Medium (DMEM, Sigma-Aldrich, St. Louis, MO, USA), supplemented with 10% fetal bovine serum (FBS, Biosciences, Princeton, NJ, USA), 2.5 mM L-glutamine, and antibiotic solution (streptomycin 100 µg/mL and penicillin 100 U/mL, Sigma-Aldrich). Cell cultures were maintained at 37 °C in a humidified atmosphere containing 5% CO_2_.

Upon reaching approximately 90% confluence, cells were trypsinized and seeded onto the membranes at a density of 3 × 10^4^ cells/mL. Cell viability was assessed at 3, 5, and 7 days using the resazurin assay (R7017; Sigma-Aldrich). At each experimental period, 200 µL of fresh culture medium and 20 µL of resazurin were added to each well and incubated for 4 h. The absorbance was quantified using a microplate reader at 600 nm (ChroMate; Awareness Technology, Palm City, FL, USA).

Cell–cell and cell–material interactions were further evaluated using a Live/Dead Cell Staining Kit (ENZO ALX-850-249). Briefly, 1 × 10^3^ cells were seeded onto the membranes and incubated for 24 h. Samples were then gently washed twice with PBS and stained using a cell-permeable green fluorescent dye (Ex/Em = 488/518 nm) to label live cells, and propidium iodide (Ex/Em = 488/515 nm) to identify dead cells. Fluorescent images were acquired using a fluorescence microscope at magnifications of 10×, 20×, and 40×.

### 2.4. Statistical Analysis

Data analysis was performed using GraphPad Prism 9 software. The Shapiro–Wilk and Bartlett tests were employed to verify data normality and variance homogeneity. To determine significant differences among groups, one-way ANOVA followed by Tukey’s post hoc analysis was conducted. All quantitative data were expressed as the arithmetic mean with standard error of the mean, with a significance level set at α < 0.05.

## 3. Results

### 3.1. FTIR and X-Ray Diffraction Analyses

In this study, polymeric WDC devices functionalized with antiseptic and analgesic components were developed. Macroscopically, all membranes exhibited a pearly white appearance and a flexible texture that allowed easy handling.

**Figure 1:**
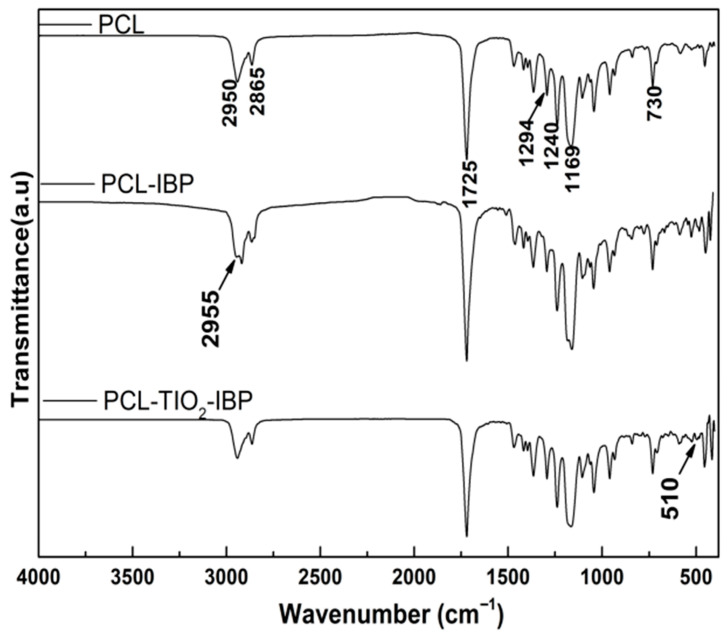
FTIR spectra. Comparative spectra of the membranes of PCL, PCL-IBP and PCL-IBP-TiO_2_ NPs are shown. PCL: polycaprolactone. IBP: Ibuprofen. TiO_2_ NPs: titanium dioxide nanoparticles.

FTIR spectra of all groups showed a characteristic absorption band at 1725 cm^−1^, corresponding to the stretching vibration of the carbonyl (C=O) group [26], which is typical of PCL (Figure 1). Additionally, asymmetric and symmetric stretching vibrations of methylene (C-H_2_) groups were observed at approximately 2950 cm^−1^ and 2865 cm^−1^, respectively [24].

In the PCL–IBP and PCL–IBP–TiO_2_ NPs membranes, the presence of IBP was confirmed by an absorption band at 2955 cm^−1^, attributed to asymmetric C-H_3_ stretching, as well as a band at 1240 cm^−1^ corresponding to C–O stretching vibrations. Furthermore, the PCL–IBP–TiO_2_ NPs group exhibited a distinct absorption band at 510 cm^−1^, which can be attributed to Ti–O–Ti stretching vibrations, indicating the successful incorporation of TiO_2_ nanoparticles [18]. No peaks corresponding to chloroform were found at 3034 cm^−1^ (C-H) or 771 cm^−1^ (C-Cl) [27].

**Figure 2:**
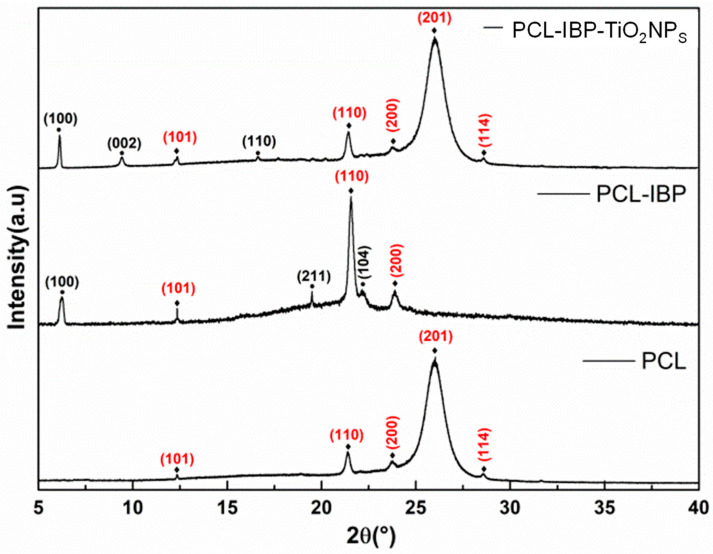
X-ray diffraction pattern. The crystallographic planes indicated with (⧫) were associated with PCL d(00-062-1286) and the crystallographic planes marked with (•) were associated with IBP (00-032-1723). PCL: polycaprolactone. IBP: Ibuprofen. TiO_2_ NPs: titanium dioxide nanoparticles.

The comparative XRD patterns of the membranes are shown in Figure 2. The PCL membrane was consistent with diffraction patterns to International Crystallography Diffraction Data (ICDD) standards PDF 00-062-1286 [28] for the composites PCL-IBP and PCL-IBP-TiO_2_ NPs. Characteristic diffraction peaks corresponding to the (101), (110), (200), (201), and (114) crystallographic planes of PCL were observed, indicating that the crystalline structure of PCL was maintained after functionalization. The presence of IBP in the composite membranes was confirmed by diffraction peaks associated with the reference pattern PDF 00-032-1723 (ICCD) [29], corresponding to the monoclinic crystalline structure of IBP, with reflections indexed to the (100), (002), (110), (211), and (104) planes.

### 3.2. Microstructural Evaluation

**Figure 3:**
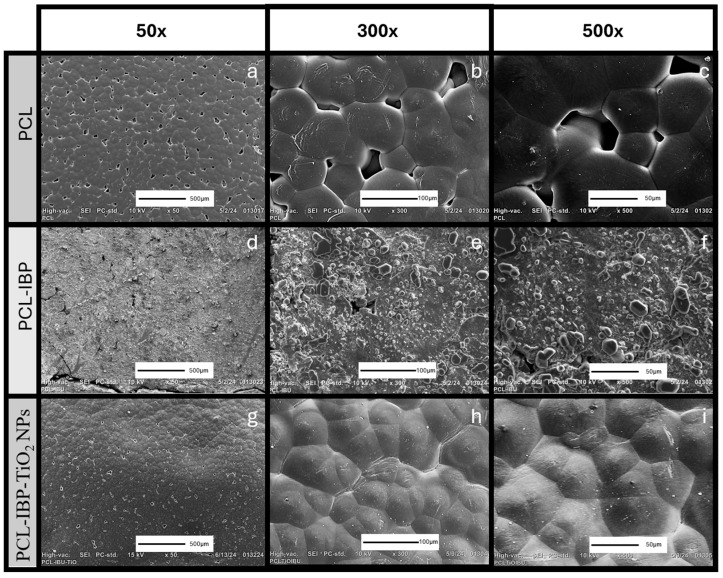
SEM Characterization. Photomicrographs of the morphology of membranes of PCL (**a**–**c**), PCL-IBP (**d**–**f**) and PCL-IBP-TiO_2_ NPs (**g**–**i**). 50×, 300×, 500× magnification. SEM: Scanning electron microscope. PCL: polycaprolactone. IBP: Ibuprofen. TiO_2_ NPs: titanium dioxide nanoparticles.

The SEM evaluation revealed distinct surface morphologies among the different membrane formulations (Figure 3). The control PCL membrane exhibited a relatively smooth and flat surface with dispersed droplet-like features and interrupted porosity (Figure 3a–c).

Incorporation of IBP resulted in noticeable changes in surface morphology. The PCL–IBP membranes displayed increased surface roughness accompanied by a reduction in pore density (Figure 3d–f). Interestingly, the PCL–IBP–TiO_2_ NPs membranes showed a more homogeneous and compact surface morphology. The presence of TiO_2_ nanoparticles appeared to reduce the agglomeration of IBP particles, resulting in the flattest surface among all groups and a further decrease in porosity (Figure 3g–i).

### 3.3. Biocompatibility Evaluation

**Figure 4:**
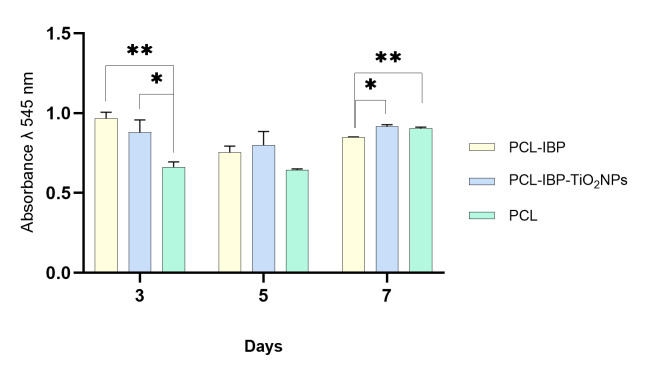
Cell viability test. The hFOB response seeded onto the surface of PCL, PCL-IBP, and PCL-IBP-TiO_2_ NPs indicates the biocompatibility of scaffolds. All data was present as mean, standard error and α < 0.05. PCL: polycaprolactone. IBP: Ibuprofen. TiO_2_ NPs: titanium dioxide nanoparticles. *: *p* < 0.05. ** *p* < 0.01.

Cell–material interaction assays indicated good biocompatibility of all membrane formulations. At day 3, both the PCL–IBP and PCL–IBP–TiO_2_ NPs groups exhibited higher resazurin absorbance values compared to the control group (Figure 4). The absorbance values of the PCL–IBP (0.965) and PCL–IBP–TiO_2_ NPs (0.879) groups were not significantly different from each other (*p* = 0.3350), while both were significantly higher than the control group (*p* = 0.0014 and *p* = 0.0491, respectively).

At day 7, no evidence of cytotoxicity was observed in any of the evaluated groups. The absorbance value of the PCL–IBP group (0.847) was lower than that of the control group (0.904; *p* = 0.007) and the PCL–IBP–TiO_2_ NPs group (0.915; *p* = 0.015). In contrast, no statistically significant difference was observed between the control and PCL–IBP–TiO_2_ NPs groups (*p* = 0.475), suggesting sustained cell viability in the presence of TiO_2_ nanoparticles.

**Figure 5:**
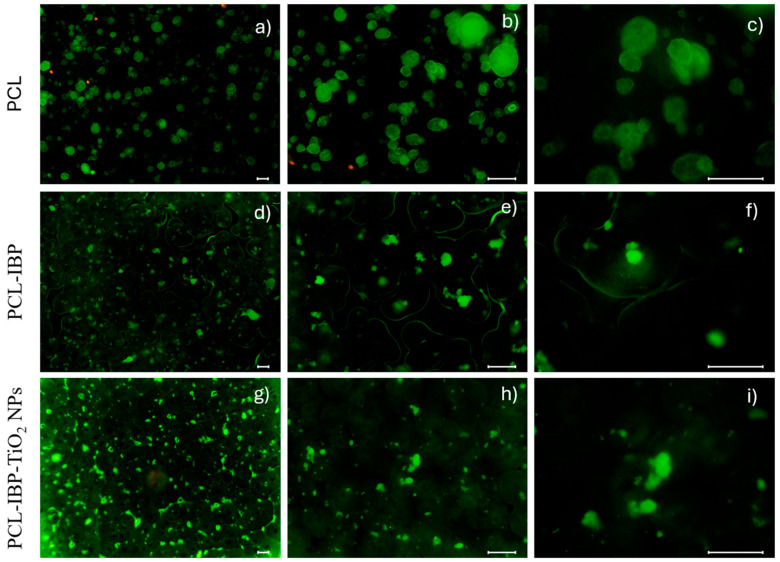
Live/Dead test. The fluorescent images of membranes at different magnifications (10×, 20×, 40×) of PCL (**a**–**c**), PCL-IBP (**d**–**f**) and PCL-IBP-TiO_2_ NPs (**g**–**i**), PCL: polycaprolactone. IBP: Ibuprofen. TiO_2_ NPs: titanium dioxide nanoparticles. Bar = 100 µm.

Live/Dead staining further supported the biocompatibility of the membranes (Figure 5). The control PCL group exhibited a higher proportion of dead (red) cells compared to the composite groups; however, live (green) cells predominated in all cases. In the PCL–IBP group, material autofluorescence outlined the scaffold perimeter and promoted the formation of cell clusters in the central region, where a high density of viable cells was observed. The PCL–IBP–TiO_2_ NPs membranes showed a more homogeneous distribution of live cells across the surface, while reduced porosity was evident due to material autofluorescence.

## 4. Discussion

In this study, polymeric WDC devices were developed using PCL-based membranes. PCL has been widely employed in medical applications due to its biodegradability, tunable mechanical properties, ease of manufacturing, and capacity to incorporate therapeutic agents [18,20,24]. These characteristics have been demonstrated in electrospun PCL-based systems functionalized with TiO_2_ and tetracycline for controlled drug delivery against Gram-positive and Gram-negative bacteria [30].

Electrospinning is one of the most commonly used techniques for WDC fabrication, as it enables the production of fibrous structures with controlled physical and chemical properties through the application of high-voltage electric fields [31]. However, this approach raises concerns related to the use of cytotoxic solvents and the requirement for high voltages. In the present work, we sought to combine the well-documented antimicrobial potential of TiO_2_ nanoparticles and the analgesic properties of ibuprofen using a manufacturing strategy that does not rely on high-voltage processing. This electric-free and straightforward fabrication method represents a simple and accessible alternative that preserves molecular integrity during membrane formation.

This assumption was supported by FTIR and XRD analyses, which indicated that the incorporation of ibuprofen and TiO_2_ nanoparticles did not induce detectable alterations in the chemical backbone or crystalline structure of PCL [18]. The preservation of characteristic PCL absorption bands and diffraction planes, together with the identification of IBP and TiO2-related signals, suggests that the rapid manufacturing approach enables the integration of bioactive components while maintaining structural stability, a critical requirement for wound dressing applications.

During solvent evaporation, polymer precipitation likely occurred in a non-uniform manner, resulting in heterogeneous polymer-rich regions and surface porosity, as observed in SEM micrographs. Such morphological features have been reported to influence cell–material interactions by modulating focal adhesion sites and the spatial organization of adhesion-related proteins such as vinculin and paxillin [32]. Accordingly, the surface variations observed in the present membranes may have contributed to the differences in cellular distribution and adhesion behavior [33].

Fluorescence imaging revealed that cells tended to cluster within polymer-rich regions or displayed a more heterogeneous distribution in coated membranes, which may explain the observed differences in metabolic activity. The increased surface roughness of the PCL–IBP and PCL–IBP–TiO_2_ membranes likely enhanced initial cell adhesion, resulting in higher metabolic activity at early time points. Taken together, these findings indicate that the incorporation of ibuprofen and TiO_2_ nanoparticles did not induce cytotoxic effects over the evaluated period. The absence of a long-term increase in metabolic activity, particularly in the PCL–IBP group, should not be interpreted as a detrimental effect, but rather as evidence of stable cell–material interactions, since the resazurin assay reflects metabolic activity rather than cell proliferation. Under these conditions, all formulations supported sustained cellular viability, fulfilling the primary objective of establishing biocompatibility of the proposed manufacturing approach.

Metallic nanoparticles have been widely explored in WDC applications due to their antimicrobial properties. For instance, PCL membranes functionalized with gelatin and silver nanoparticles have shown dose-dependent inhibition of bacterial growth [34]. In the case of TiO_2_ nanoparticles, their photoactivity under UV exposure leads to the generation of reactive oxygen species, suggesting potential antimicrobial functionality in wound environments exposed to light [35]. Additionally, the incorporation of ibuprofen may contribute to localized anti-inflammatory and analgesic effects without systemic exposure. Although these functional properties were not directly evaluated in the present study, the results suggest that the proposed membranes constitute a promising platform for future multifunctional wound healing applications.

## 5. Conclusions

The present study describes the development of a PCL-based WDC with several potential advantages. First, the absence of high-voltage processing offers a safer and simpler manufacturing approach compared with electrospinning-based techniques. Second, the membranes were readily functionalized with ibuprofen and TiO_2_ nanoparticles while preserving molecular integrity, as confirmed by spectroscopic analyses. Third, the integration of bioactive components with reported antimicrobial and anti-inflammatory potential suggests that the proposed system may serve for wound care applications. Finally, the photoactive properties of TiO_2_ nanoparticles further support the suitability of these membranes for external skin injuries. Overall, the developed WDCs demonstrated manufacturing stability and favorable biocompatibility with hFOB cells, fulfilling the objective of establishing a safe and structurally stable platform for future wound healing applications.

## Data Availability

The original contributions presented in this study are included in the article. Further inquiries can be directed to the corresponding author.

## Abbreviations

The following abbreviations are used in this manuscript:

WDC	Wound Dressing Coverage
NSAID	Non-Steroidal Anti-Inflammatory
PCL	Polycaprolactone
NPs	Nanoparticles
TiO_2_	Titanium Dioxide
IBP	Ibuprofen
FTIR	Fourier Transform Infrared Spectroscopy
XRD	X-ray Diffraction
SEM	Scanning Electron Microscopy
